# The Importance of Preoperative NLR, PLR, and MPV Values in Predicting the Risk of Complications in Colorectal Peritoneal Carcinomatosis

**DOI:** 10.3390/jpm14090916

**Published:** 2024-08-29

**Authors:** Pırıltı Özcan, Özgül Düzgün

**Affiliations:** 1 Department of General Surgery, Cerrahpaşa Faculty of Medicine, Istanbul University, 34098 Istanbul, Turkey; 2 Department of Surgical Oncology, İstanbul Umraniye Training and Research Hospital, University of Health Sciences, 34766 Istanbul, Turkey; ozgul.duzgun@sbu.edu.tr

**Keywords:** NLR, PLR, MPV, colorectal, peritoneal carcinomatosis

## Abstract

Background: Colorectal cancer peritoneal carcinomatosis (CRC PC) necessitates preoperative assessment of inflammatory markers to predict postoperative outcomes and guide treatment. This study aims to evaluate the prognostic value of preoperative Neutrophil–Lymphocyte Ratio (NLR), Platelet–Lymphocyte Ratio (PLR), and Mean Platelet Volume (MPV) in predicting complications for CRC PC patients undergoing surgery. Methods: Calculating NLR, PLR, and MPV from patient data: NLR = absolute neutrophil count/total lymphocyte count, PLR = total lymphocyte count/total platelet count × 100, and MPV = platelet crit (PCT)/total platelet count. Result: The study included 196 CRC PC patients and found significant relationships between these markers and overall survival (OS). Patients with an NLR of 3.77 had a median OS of 22.1 months, compared to 58.3 months for those with lower NLR (HR 2.7, 95% CI 1.1–5.3, *p* < 0.001). Conclusions: For CRC PC patients undergoing CRS+HIPEC, preoperative assessment of NLR, PLR, and MPV can serve as independent prognostic markers for OS. Incorporating these markers into preoperative evaluations may improve patient selection and outcome prediction.

## 1. Introduction

Peritoneal dissemination due to intra-abdominal malignancies is defined as stage 4 disease and diagnosed as peritoneal carcinomatosis (PC). PC is considered a terminal illness with low survival rates. PC related to colorectal cancer (CRC PC) has been reported in literature with rates up to approximately 13% [1,2,3,4]. CRC PC is observed synchronously in about 7% of cases and metachronously in about 6% of cases [5,6]. PC is associated with decreased overall survival (OS) and significantly worse prognosis—approximately 30–40% of cases compared to non-peritoneal metastases [7,8,9].

CRC PC has become a promising treatment with cytoreductive surgery (CRS) and hyperthermic intraperitoneal chemotherapy (HIPEC), achieving up to 38% five-year OS rates in selected cases with a low peritoneal carcinomatosis index (PCI) [10,11,12,13,14,15,16].The PCI score is one of the most important prognostic parameters in selecting patients who will benefit from CRS+HIPEC. Calculating the PCI score preoperatively helps prevent unnecessary surgeries and is crucial in identifying patients who will benefit from aggressive surgery and determining prognosis [17,18,19]. A PCI score can be calculated using radiological imaging methods such as CT, MRI, and PET CT, and also through diagnostic laparoscopic methods. However, these methods are expensive and invasive. Therefore, for advanced cancer patients with CRC PC undergoing CRS+HIPEC, there is a need for cheaper, easily accessible, and measurable prognostic biomarkers in the preoperative period to predict postoperative outcomes and even guide treatment decisions.

Researchers in recent years have focused on studies questioning the potential of inflammatory biomarkers to act as prognostic indicators showing inflammatory status in various types of cancer [20]. Excessive inflammation leads to immune system imbalance, tumor cell growth, and reduced survival [21,22]. Neutrophils (N), lymphocytes (L), platelets (P), and monocytes (M) are significant biomarkers indicating inflammation. Measurements such as the neutrophil-to-lymphocyte ratio (NLR), the platelet-to-lymphocyte ratio (PLR), and mean platelet volume (MPV) are inexpensive approaches; moreover, these values can be easily assessed in most hospital laboratories, and these markers have been reported to be significantly associated with the prognosis of cancers including PC [23,24,25,26,27,28,29]. There is limited data in the literature regarding the prognostic value of NLR, PLR, and MPV in patients undergoing CRS+HIPEC due to CRC PC. While research in the preoperative period is very inadequate, publications generally focus on the postoperative period [30,31,32,33,34,35].

The Clavien–Dindo (CD) classification is a widely used system for categorizing surgical complications based on their severity [36]. It was first proposed by Clavien and Dindo in 2004 and has since become a standard for reporting and comparing surgical outcomes. The CD classification aims to standardize the reporting of postoperative complications and facilitate comparison of surgical outcomes across different studies and institutions. It also provides a standardized way to evaluate surgical quality and report complications, categorizing them based on severity and required interventions. This enhances the understanding and management of postoperative outcomes, ultimately contributing to improving patient care and surgical practices.

The aim of our study is to highlight the significance of preoperative inflammatory parameters such as NLR, PLR, and MPV in predicting postoperative complications in patients undergoing surgery due to CRC PC.

## 2. Patients and Methods

### 2.1. Patient Information and Ethical Considerations

Between October 2016 and January 2024, data from patients surgically treated for CRC PC at the Surgical Oncology Clinic of Health Sciences University Ümraniye Training and Research Hospital were collected prospectively and evaluated retrospectively from the electronic record system. The study was conducted with approval from the ethics committee of Ümraniye Training and Research Hospital under study number 2024/58. The study included cases aged 18–70 years with a diagnosis of CRC, without a history of surgery or infection within 15 days, who underwent CRS+HIPEC due to CRC PC. Patients requiring emergency surgery due to ileus, those who underwent palliative surgery, those considered inoperable, those with active infection history within the first 15 days preoperatively, and those with incomplete preoperative blood values were excluded from the study. Diagnostic laparoscopy was performed for all cases before CRS. CRS was performed using the surgical technique described by Sugarbaker, and postoperative HIPEC involved MMC 35 mg/Body Surface Area (BSA) for 60 min at 43 degrees Celsius.

Patients’ demographic data, tumor locations, PCI score, completeness of cytoreduction score (CC), and preoperative blood parameters taken within 7 days prior to surgery (total white blood cell count (WBC), N, L, M, P, NLR, PLR, and MPV) were collected. NLR, PLR, and MPV were calculated using the following formulas: NLR = (absolute neutrophil count)/(total lymphocyte count), PLR = (total lymphocyte count)/(total platelet count) × 100, MPV = mean platelet volume calculated by dividing platelet crit (PCT) by total platelet count. Complications of CD Grade 3 and above occurring within the first 30 days postoperatively were recorded. Additionally, progression-free survival (PFS) and OS data were also collected.

### 2.2. Statistical Analyses

All statistical analyses were conducted using SPSS version 25.0 (SPSS Inc., Chicago, IL, USA). The area under the ROC curve (AUC) was calculated as a measure of the ability of each potential biomarker to discriminate between two groups. For biomarkers showing statistically significant ROC curves, the relationship between each biomarker and clinical-pathological variables was evaluated. In order to analyze the data, due to practical reasons 75% percentile was used. Continuous variables were presented as mean ± SD (95% CI) and compared between groups using Student’s *t*-test to assess variance. Categorical variables were presented as numbers and percentages, and a chi-square test or Fisher’s exact test was used as appropriate. Pearson correlation analysis was used to examine correlations between variables. Statistical significance was set at *p* < 0.05. For the survival modeling technique, the Kaplan Meier curve was used.

## 3. Results

A total of 216 patients underwent surgery due to CRC PC. After excluding 20 cases due to high PCI scores or the need for palliative surgery, a total of 196 cases were included in the study (Table 1 and Table 2).

CD Grade 3 and above complications developed in 22 cases (11.4%). Ten cases (5%) required reoperation due to anastomotic leakage. Additionally, pleural effusion occurred in four cases (2%), intra-abdominal ascites in three cases (1.5%), hemofiltration due to renal failure in two cases (1%), and one case each of cardiac arrhythmia, pulmonary embolism, and COVID-19 infection. Among these 22 cases, mortality occurred in eight cases. The median DFS was 16 months and OS was 58 months, with a 5-year OS rate of 46% (Table 3).

A statistically significant association was observed between NLR and CD complications, with patients having an NLR at the 75th percentile (3.77) experiencing a 20% rate of Grade 3 complications compared to 8% in those with an NLR below 3.77 (HR 2.6, 95% CI 1.2–5.4, *p* < 0.01). Additionally, NLR was linked to overall survival (OS), where patients at the 75th percentile had a median OS of 22.1 months, significantly shorter than the 58.3 months for those with lower NLR values (HR 2.7, 95% CI 1.1–5.3, *p* < 0.01). Although there was no significant association with disease-free survival (DFS), multivariate analysis confirmed NLR as an independent predictor for both DFS (HR 2.3, 95% CI 1.3–4.2, *p* < 0.01) and OS (HR 5.5, 95% CI 2.6–11.7, *p* < 0.01). PLR also showed a significant association with CD Grade 3 complications, with a 10% complication rate at the 75th percentile (150) compared to 21% below this value (HR 1.9, 95% CI 1.05–3.55, *p* = 0.03). PLR was an independent predictor for OS (HR 2.0, 95% CI 1.03–3.9, *p* < 0.05) and DFS (HR 2.0, 95% CI 1.03–3.9, *p* < 0.05), with a median DFS of 22 months for patients at the 75th percentile compared to 18 months for those below. MPV was similarly associated with CD Grade 3 complications and OS, with an 18% complication rate at the 75th percentile (8) versus 9% below (HR 1.8, 95% CI 1.07–3.75, *p* = 0.03), and a median OS of 40 months compared to 17 months below this value (HR 1.8, 95% CI 1.1–3.31, *p* = 0.03). MPV was also confirmed as an independent predictor for DFS in multivariate analysis (HR 1.9, 95% CI 1.06–3.8, *p* < 0.05) (Table 4, Table 5, Table 6 and Table 7).

## 4. Discussion

In the past three decades, CRS+HIPEC has become widely used in CRC PC and has shown promising results in selected cases with low PCI scores and high CC 0 rates, as evidenced by a randomized clinical trial comparing it with systemic chemotherapy (median survival: 22.3 months vs. 12.6 months, *p* = 0.032) [37]. Subsequent studies have reported median survival times ranging from 13 to 63 months [38,39,40,41,42]. PCI and CC scores have been reported as the most important factors associated with survival, helping to identify patients who benefit from these surgeries [43].

Various scoring systems are available to evaluate CRC PC patients prognostically, preoperatively, and perioperatively. However, these scoring systems often require expensive and invasive procedures such as radiological imaging methods and diagnostic laparoscopies. Therefore, in recent years, many studies have focused on analyzing biochemical and hematological markers in the preoperative period to study interactions between tumor cells and the immune system [43,44,45,46]. Nevertheless, the role of serological biomarkers in predicting the prognosis of CRC PC patients is limited, and there are few studies investigating the clinical benefits of biomarkers [35]. Markers such as NLR, PLR, and MPV are cost-effective and can be easily performed using routine laboratory analysis. Consequently, these markers have started to be used as new inflammation markers in various cancer types [47,48,49,50].

CRS is a complex and invasive surgical technique that, despite improvements in experience over time leading to reduced complication rates, still presents higher risks compared to standard surgical operations. Common complications include anastomotic leaks, bleeding, and infections, as well as renal, cardiac, pulmonary, neurological, and thromboembolic issues [51]. In a phase 2 randomized clinical trial by Rovers et al. comparing perioperative systemic therapy with CRS+HIPEC in 79 cases of CRC PC, they found postoperative morbidity of CD Grade 3 or higher to be 22% versus 33% in patients undergoing CRS+HIPEC [44]. Livin et al., in a single-center retrospective study, reported a rate of CD Grade 3 complications at 23.2%, with Grade 4 complications at 5.1%, and Grade 5 complications in 2% of cases [19]. According to Halkia et al. mortality rates ranging from 0–10% and morbidity rates from 0–62% were observed in 1069 cases of mixed etiology PC, with an inoperability rate reported at 17.1% [52]. In our study, we initially observed a rate of CD Grade 3 or higher complications of around 25%, which decreased with experience to an overall rate of 11.4%.

The NLR, PLR, and MPV can be easily obtained from routine preoperative complete blood counts. If these markers demonstrate prognostic significance, they can potentially improve patient selection for CRS and HIPEC [47]. Studies have shown that NLR is a useful prognostic tool in predicting DFS and OS in cancer patients [53,54]. In a retrospective cohort series by Zager et al., including 105 CRS-HIPEC procedures in 98 patients, they found that nearly one-fifth of CRS cases were inoperable. They also identified that preoperative NLR and PLR were not associated with operability, but high preoperative NLR was correlated with worse median OS [35]. Li et al. demonstrated the adverse impact of initial NLR on both OS and PFS in CRC patients [49]. Similarly, Rangarajan et al. showed that higher NLR was associated with worse DFS and OS in patients with appendiceal pseudomyxoma peritonei (PMP) [30]. In our study, NLR emerged as a strong, independent prognostic factor in our patient population. Patients with an NLR ≤ 3.567 had a median DFS and OS of 15 and 55.5 months, respectively, whereas those with an NLR > 3.567 had significantly shorter median DFS (9.8 months) and OS (26.4 months), nearly twice as low. This finding is consistent with existing literature and is supported by the differential roles of neutrophils and lymphocytes in host immune response, as discussed above.

PLR has been shown to be an independent risk factor in cancer patients through various studies [35]. In CRC, there is evidence of an inverse correlation between preoperative PLR values and OS [47,48,49,50,51,52,53,54,55]. Templeton et al., in a meta-analysis involving 12,754 patients, investigated the relationship between PLR and OS across various solid tumors and concluded that PLR is independently associated with OS [34]. Similarly, Loh et al. identified PLR as an independent negative prognostic factor for OS in a series of 144 patients undergoing CRS/HIPEC [56]. In our own study, we also found that NLR, PLR, and MPV are independent prognostic factors.

In CRC with PC, the inflammatory role of MPV levels has been less explored in the literature compared to NLR and PLR. The only publication on this topic, by Kim et al., conducted a retrospective study of 160 cases. They investigated the potential relationship between preoperative MPV and postoperative prognosis in PC patients undergoing CRS with HIPEC. They found that higher preoperative MPV was associated with a median OS of 36 months (95% CI, 26.6–45.4) and reported a 5-year OS rate of 40.5% (27.3–51.6%). They also observed that patients with increased MPV had lower 1-year survival rates [57]. In our study, we observed a similar trend: patients with MPV values at the 75th percentile (p75, 8) had a median OS of 40 months, whereas those with MPV values below 8 had a median OS of 17 months. This finding is consistent with the results reported by Kim et al. (HR 1.8, 95% CI 1.1–3.31, *p* = 0.03). Herold et al., in their retrospective observational study of 835 colorectal cancer patients, demonstrated that patients’ comorbidities and previous surgeries affected their blood test results [58]. Controlled comorbidities are important in assessing better CBC counts. This is a limitation in our study since we have not taken into consideration comorbidities and previous surgeries.

Our study identified several limitations including its retrospective, single-center design and small sample size. Therefore, it is necessary to investigate these markers in larger prospective and randomized trials. Despite these limitations, we believe that our study convincingly demonstrates the prognostic impact of preoperative PLR, NLR, and MPV in predicting complications and estimating OS in patients undergoing CRS and HIPEC for CRC PC. Therefore, we recommend considering these markers alongside clinical and radiological factors in the preoperative selection of patients undergoing this procedure.

## 5. Conclusions

In cases of CRC with PC, NLR, PLR, and MCV examined during the preoperative period there are independent prognostic markers for OS in the planning of CRS+HIPEC. A statistically significant association was observed between NLR and CD complications, with patients having an NLR at the 75th percentile (3.77) experiencing a 20% rate of Grade 3 complications compared to 8% in those with an NLR below 3.77 (HR 2.6, 95% CI 1.2–5.4, *p* < 0.01). In conclusion, the integration of CBC and HIPEC represents an advancement in the treatment strategy for peritoneal carcinomatosis. This study not only underscores the efficacy of this combined approach but also sets the stage for future research aimed at optimizing and personalizing cancer treatment protocols. The novelty of this research lies in its potential to enhance patient outcomes through innovative therapeutic combinations. We recommend their consideration in predicting complications during the preoperative period and therefore in patient selection.

## Figures and Tables

**Table 1:** Baseline demographic data.

Variables	Total (n = 196)
Men (%)	112 (57.1%)
Age (year)	
Men	57.3 (27–74)
Women	56.9 (29–72)
Tumor origin	
Colon	152 (77.5%)
Rectum	44 (22.4%)
Tumor site	
Right colon	68 (44.7%)
Transverse colon	13 (8.5%)
Left colon + sigmoid	71 (46.7%)
Histological classification	
Serous adenocarcinoma	140 (71.4%)
Mucinous adenocarcinoma	49 (25%)
Signet-ring cell carcinoma	4 (2%)
Adenosquamous carcinoma	3 (1.5%)
Neoadjuvant Therapy	153 (78%)

Data are presented as median (interquartile range) or n (%).

**Table 2:** Perioperative findings.

Variables	Total (n = 196)
PCI Score	8 (3–18)
CC Score 0/1	189 (96.4%)
Operation time (minute)	420 (185–600)
Blood loss (mL)	550 (200–4200)
FFP transfusion (unit)	1.2 (0–7)
Whole blood transfusion (unit)	2 (0–9)
Urine output (mL)	800 (400–3500)
Hospitalization Time (day)	8 (7–45)
Complication *	22 (11.2%)
Reoperation due to anastomosis leakage	10 (5.1%)
Pleural effusion	4 (2%)
Intraabdominal ascites	3 (1.5%)
Hemofiltration due to renal failure	2 (1%)
Cardiac arrhythmia	1 (0.5%)
Pulmonary embolism	1 (0.5%)
COVID-19	1 (0.5%)
Mortality	8 (4.1%)
DFS (month)	16
OS (month)	58
5-year survival rate	46%

Data are presented as median (interquartile range) or n (%), PCI: Peritoneal Cancer Index, CC: Completeness of Cytoreduction, FFP: Fresh Frozen Plasma, DFS: Disease-Free Survival, OS: Overall Survival, * Clavien–Dindo Grade 3 and higher.

**Table 3:** Preoperative blood values.

	Total (n = 196)
Leukocyte (109/L)	6.6 (4.2–8.9)
Hemoglobin (g/L)	11 (97–140)
Platelet (109/L)	310,000 (190,000–579,000)
Lymphocyte (109/L)	1.68 ± 49
PLR	150 (77–378)
NLR	2.38 (3.01–21.6)
MPV (fL)	8.09 (7.33–8.60)

Data are presented as mean ± SD or median (interquartile range), PLR: Platelet–lymphocyte ratio, NLR: Neutrophil–lymphocyte ratio, MPV: Mean Platelet Volume.

**Table 4:** NLR, PLR, and MPV correlation with Grade 3 CD, OS and DFS (in months) on univariate analysis using the Cox model and patients survival in months.

	NLR		PLR		MPV	
	<3.77	≥3.77	<190	≥190	<8	≥8
Grade 3 Complication	8%	20%	21%	10%	9%	18%
Disease-Free Survival	-	-	18	22	-	-
Overall Survival	58.2	22.1	-	-	40	17

NLR: Neutrophil–lymphocyte ratio, PLR: Platelet–lymphocyte ratio, MPV: Mean Platelet Volume, CD: Clavien–Dindo, OS: Overall Survival, DFS: Disease-Free Survival.

**Table 5:** Univariate analyses of factors affecting CD Grade 3 Complication of patients with peritoneal carcinomatosis.

	Univariate Analysis		
	HR	95%CI	*p* Value
NLR (<3.77 vs. ≥3.77)	2.6	1.2–5.4	<0.01
PLR (<150 vs. ≥150)	1.9	1.05–3.55	<0.03
MPV (<8 vs. ≥8)	1.8	1.07–3.75	<0.03

CD: Clavien–Dindo, NLR: Neutrophil–lymphocyte ratio, PLR: Platelet–lymphocyte ratio, MPV: Mean Platelet Volume, HR: Hazard Ratio.

**Table 6:** Univariate and multivariate analyses of factors affecting DFS of patients with peritoneal carcinomatosis.

	Univariate Analysis			Multivariate Analysis		
	HR	95%CI	*p* Value	HR	95%CI	*p* Value
NLR (<3.77 vs. ≥3.77)	-	-	-	2.3	1.3–4.2	<0.01
PLR (<150 vs. ≥150)	1.9	1.06–3.54	0.03	2.0	1.03–3.9	<0.05
MPV (<8 vs. ≥8)	-	-	-	1.9	1.06–3.8	<0.05

DFS: Disease-Free Survival, NLR: Neutrophil–lymphocyte ratio, PLR: Platelet–lymphocyte ratio, MPV: Mean Platelet Volume, HR: Hazard Ratio.

**Table 7:** Univariate and multivariate analyses of factors affecting OS of patients with peritoneal carcinomatosis.

	Univariate Analysis			Multivariate Analysis		
	HR	95%CI	*p* Value	HR	95%CI	*p* Value
NLR (<3.77 vs. ≥3.77)	2.7	1.1–5.3	<0.01	5.5	2.6–11.7	<0.01
PLR (<150 vs. ≥150)				2.0	1.03–3.9	<0.05
MPV (<8 vs. ≥8)	1.8	1.1–3.31	<0.03			

OS: Overall Survival, NLR: Neutrophil–lymphocyte ratio, PLR: Platelet–lymphocyte ratio, MPV: Mean Platelet Volume, HR: Hazard Ratio.

## Data Availability

The original contributions presented in the study are included in the article further inquiries can be directed to the corresponding authors.
